# Effects of viral infection on passion fruit (*Passiflora edulis*) quality and productivity in the Republic of Korea

**DOI:** 10.3389/fpls.2025.1612094

**Published:** 2025-06-19

**Authors:** Minkyung Choi

**Affiliations:** Fruit and Vegetable Research Institute, Jeonbuk State Agricultural Research & Extension Services, Iksan, Republic of Korea

**Keywords:** cucumber mosaic virus, East Asian Passiflora virus, Euphorbia leaf curl virus, papaya leaf curl Guandong virus, plant viral diseases

## Abstract

Passion fruit (*Passiflora edulis*) is a widely cultivated plant in the Republic of Korea, but its cultivation is impacted by a variety of viral diseases. This study analyzed viral diseases in *P. edulis* and their effects on fruit quality and productivity. Surveys were conducted in a major *P. edulis*-producing region in the Republic of Korea from 2019 to 2022, and viral infection experiments were performed in a greenhouse test plot. The main viruses detected were East Asian passiflora virus (EAPV), papaya leaf curl Guandong virus (PaLCuGdV), cucumber mosaic virus (CMV), and Euphorbia leaf curl virus (EuLCV). Viral infection incidence increased as cultivation years increased, with EAPV showing the highest rate at five years. EAPV had the highest infection rate during the fruit enlargement stage, while CMV peaked in the early growth and flowering stages. Fruit from virus-infected *P. edulis* exhibited lower quality, with reduced Brix, titratable acidity, and fruit number. EAPV caused the most severe effects. The study highlights that uninfected *P. edulis* maintained stable productivity, demonstrating the potential for extending cultivation beyond the typical five-year period with effective disease management strategies. This underscores the importance of robust viral disease management to sustain long-term productivity in the *P. edulis* industry. This study provides foundational data to enhance the stability of *P. edulis* cultivation and prevent the spread of viral diseases, which could improve the sustainability of the industry and bolster economic resilience for farms.

## 1 Introduction

Passion fruit is a perennial vine in the family Passifloraceae, native to Brazil and South America (Bernacci et al., 2008; Colariccio et al., 2020). Depending on the color of the skin, passion fruit is divided into purple (*Passiflora edulis* Sims) and yellow (*P. edulis* f. *flavicarpa*) (Cazarin et al., 2016; Deng et al., 2010) varieties. Globally, approximately 1.4 million tonnes of passion fruit are produced each year, with Brazil being the biggest producer, at 600,000 tonnes (Altendorf, 2018). Passion fruit is an important economic crop in tropical and subtropical regions, especially in South America (Huang et al., 2021; Kawakami et al., 2022).

*P. edulis* fruit is well known for its unique flavor, is rich in several nutrients, including amino acids and minerals, and provides various health benefits, including anti-inflammatory, antioxidant, and anti-cancer effects (He et al., 2020; Fonseca et al., 2022; Teng et al., 2022). *P. edulis* was introduced to the Republic of Korea in 1989, and since it began to be cultivated commercially in 2012, demand for it has grown due to its unique taste and effects, accompanied by nationwide expansion of the cultivation area (Jeon et al., 2022; Park et al., 2024). Due to the effects of climate change causing global warming, cultivation of sub-tropical crops has been increasing in the Republic of Korea, especially in the southern region. For example, the cultivation area for sub-tropical fruit trees was 1,024,000 m^2^ for nine species in 2017, and this increased to 1,713,000 m^2^ for ten species in 2021 (Rural Development Administration, 2023).

The cultivation and fruit production of passion fruit is affected by various pathogens, including bacteria, molds, and viruses (Cubero et al., 2016). The main bacterial disease that has been reported globally is bacterial spot due to *Xanthomonas axonopodis* pv. *passiflorae*, which has been observed in Australia (Bradbury, 1986), Colombia (Castilho and Granada, 1995), and Brazil (Malavolta, 1998). Mold diseases damage roots, stems, leaves, flowers, and fruit of passion fruit from the sapling stage to the mature plant stage. Several types of pathogenic mold cause severe losses in the processes of fruit storage, transport, and commercialization. The main diseases affecting the aboveground parts are anthracnose (Anaruma et al., 2010), scab (Wilingham et al., 2002), septoriosis, and Alternaria spot, while the most difficult diseases from a prevention standpoint are those that occur belowground, such as fusarium wilt, collar rot, and crown rot (Fischer and Rezende, 2008).

Viral diseases pose a very serious risk for *P. edulis* cultivation, and are major factors resulting in decrease in fruit production. As a perennial crop, when *P. edulis* is infected by a virus, productivity and fruit quality decline continuously throughout the cultivation period, and plant vitality also decreases, leading to massive losses for farms (Wu et al., 2024). *P. edulis* can be infected by viruses via several routes, and more than 30 viral species have been found to date (Huang et al., 2023). The viruses affecting these plants are categorized into several genera: *Potyvirus*, which includes the East Asian Passiflora virus (EAPV) (Iwai et al., 2006), Telosma mosaic virus (TeMV) (Chiemsombat et al., 2014), and passion fruit woodiness virus (PWV) (Sokhandan et al., 1997); *Carlavirus*, represented by Passiflora latent virus (PLV) (Spiegel et al., 2007); Begomovirus, which encompasses viruses such as passion fruit severe leaf distortion virus (PSLDV) (Ferreira et al., 2010), Euphorbia leaf curl virus (EuLCV), papaya leaf curl Guangdong virus (PaLCuGdV) (Cheng et al., 2014), and papaya leaf curl China virus (PaLCuCNV) (Huang et al., 2020); and *Cytorhabdovirus*, which includes citrus-associated rhabdovirus (CiaRV) (Zhang et al., 2021).

Huang et al. (2023) reported that TeMV, EAPV, CiaRV, and PLV have mostly been detected in *P. edulis* in China. Cheng et al. (2014) first reported EuLCV and PaLCuGdV in passion fruit, and these were the first cases in Taiwan and in Asia. According to a report by Hong (2017), of the 30 viruses affecting passion fruit, cucumber mosaic virus (CMV), EAPV, EuLCV, PaLCuGdV, and tomato yellow leaf curl virus (TYLCV) have been detected in *P. edulis* in the Republic of Korea. Of these, EuLCV, PaLCuGdV, and EAPV were confirmed to be infecting *P. edulis* in the Republic of Korea for the first time. Jeon et al. (2022) and Choi (2025) studied infection rates for six types of viruses, including PWV, in *P. edulis* in the Republic of Korea but did not find any infection by TYLCV. Therefore, the main viral diseases of *P. edulis* in the Republic of Korea appear to be CMV, EAPV, PaLCuGdV, and EuLCV. These viruses are usually transmitted by vectors, cutting of infected saplings, or mechanical contact. Vector-mediated infection is especially known to be transmitted via plants in the family Solanaceae as intermediate hosts (Fischer and Rezende, 2008). The symptoms of viral infection mostly consist of chlorotic spots, yellow mosaic patterns, and leaf curl. In fruits, viral infection causes severe impairment of yield and fruit quality due to corking and malformed fruit (Do et al., 2021).

We reviewed recent trends in research on the reporting, diagnosis, and prevention of pathogenic viruses in passion fruit. There have been reports of the first cases of PLV infection in *P. edulis* in the Republic of Korea and China (Bao et al., 2023; Choi and Ju, 2023), and the first report of passionfruit green spot virus in *P. edulis* f. *flavicarpa* in Colombia (Roy et al., 2023). Most research in this area has been on the progress of research on viral diseases of *P. edulis* (Wu et al., 2024), the occurrence and distribution of major viruses (Fu et al., 2023), incidence rates, and mixed viral infections in Colombia (Ceballos-Burgos et al., 2024), and simultaneous diagnosis of 4 types of viruses using multiplex RT-PCR (Huang et al., 2023). Recent studies in the Republic of Korea have reported on the incidence of six viral diseases in *P. edulis* (Jeon et al., 2022) and have analyzed the correlation between virus infections in *P. edulis* and surrounding weeds (Choi, 2025). These studies have focused on the first reports of viruses, as well as diagnostic methods and the distribution of viral diseases, using multiplex RT-PCR (Huang et al., 2023), as well as diagnostic methods and the distribution of viral diseases.

As an introduced crop, *P. edulis* in the Republic of Korea is often cultivated by agricultural workers who lack specialized knowledge or skills for handling this crop. In addition, there are no systematically prepared manuals on the major types of viral diseases or methods for prevention and management. In the field, *P. edulis is* usually propagated through cutting and grafting, and since many saplings are infected with viruses, there are concerns regarding the spread of viruses and increased damage. Viral infection causes a deterioration in fruit quality and production, but there has been a shortage of research meticulously analyzing the damage caused by these viruses by time of infection and cultivation year, or investigating management techniques to prevent their recurrence.

This study aimed to investigate the occurrence, incidence rates, and periods of infection of major viruses in *P. edulis* plantations, and to improve awareness of viral diseases of *P. edulis* among agricultural workers. In addition, we analyzed the correlation between cultivation years (age) of *P. edulis* and incidence rates, to provide useful information about the times for growth management and sapling replanting of *P. edulis*. We analyzed the effects of viral infection on the fruit quality characteristics of *P. edulis*, in order to provide information for the establishment of strategies for disease prevention and stable production of high-quality fruit. In this study, we precisely investigated viral disease-induced factors impairing fruit quality and productivity during *P. edulis* cultivation. We present management strategies based on our findings, thereby supporting the stable production of high-quality *P. edulis* fruit. We anticipate that our study will improve the sustainability of the *P. edulis* industry, help promote economic stability for farms, and, internationally, provide guidelines that can be applied in similar agricultural environments.

## 2 Materials and methods

### 2.1 Selection of study sites and analysis of the state of viral disease occurrence

To analyze the major viral diseases affecting *P. edulis* and their incidence by cultivation year, we conducted surveys in Jeonbuk State, a major *P. edulis*-producing region in the Republic of Korea. Ten species of sub-tropical fruit trees are cultivated in the Republic of Korea across an area of 1,713,000 m^2^, of which *P. edulis* accounts for the second largest area, after mangoes, of 348,000 m^2^. Jeonbuk State is the second largest grower of *P. edulis* in the Republic of Korea, with the cultivation area of 91,400 m^2^ being the largest of the 133,000 m^2^ total cultivation area for sub-tropical fruit trees in the region (Rural Development Administration, 2023). Although it is not the optimal region for cultivating *P. edulis*, Jeonbuk State has proactively introduced greenhouse cultivation techniques to enhance farm income. Consequently, within Jeonbuk State, *P. edulis* is mostly cultivated in Kimje, Namwon, Iksan, Wanju, and Jeongeup, and is being established as a new income-generating crop (Rural Development Administration, 2023). Therefore, for the survey, between 2019 and 2021, we arbitrarily selected ten farms with *P. edulis* greenhouses in Namwon, Wanju, Iksan, Kimje, and Jeongeup, where farms are densely placed.

The survey was conducted by visiting the farms at two-month intervals (February, April, June, August, October, and December). For the survey method, we arbitrarily selected three sites in each plot, and randomly selected 100 P*. edulis* plants from each site. We directly inspected each specimen showing signs of viral disease in the field and calculated the incidence rates. We then sampled the diseased parts (leaf, flower, fruit) to identify the pathogen. Based on the results of three years of surveys, we performed a correlation analysis of the incidence rates with the *P. edulis* cultivation years (age), to explore patterns of disease occurrence and contributing factors. We performed correlation analysis on the four viral species that regularly appeared during the survey period (EAPV, CMV, PaLCuGdV, and EuLCV). To ensure reliability, we excluded PLV from the analysis, which only appeared in 2021. For the correlation analysis, we used Pearson’s correlation coefficients and IBM SPSS Statistics Ver. 21.0 (IBM Co., Armonk, NY, USA).

### 2.2 Virus diagnosis and identification

For virus diagnosis, we used the methods of polymerase chain reaction (PCR) and reverse transcription polymerase chain reaction (RT-PCR). We collected 0.1 g of leaf tissue for nucleic acid extraction, placed the sample in a 1.5 ml Eppendorf tube, and homogenized the sample in liquid nitrogen. We then used a Viral Gene-spin^™^ Viral DNA/RNA Extraction Kit (iNtRON Biotechnology, Seongnam, Republic of Korea) to extract the total DNA/RNA. The purity and concentration of the extracted nucleic acids were measured using a NanoDrop spectrophotometer (Thermo Fisher SCIENTIFIC, Seoul, Republic of Korea). The nucleic acids were then divided into small portions by pipetting and stored in a deep freezer at –80°C until subsequent experiments. Viral infection was diagnosed using the specific primers described by Hong (2017) in the identification of the four species of EuLCV, PaLCuGdV, CMV, and EAPV (**Table 1**).

**Table 1:** A list of viruses and their specific diagnostic primers used in this experiment.

Virus	Primer	Primer sequences (5′–3′)	Size (bp)	Accession No. referred
EuLCV	EuLCV-F	AGTGGTCCCCCCTCCACTAAC	339	AJ811911.1
	EuLCV-R	CAGCCTCCGTCGAACCTTCG		
PaLCuGdV	PaLCuGdV-m-2F	CTGTCTTACGTGCAAGGA	605	MZ130299.1
	PaLCuGdV-m-2R	GCTTGCATATTGACCACCAG		
CMV	CMV 1-5 u	CTTGTGCGTTTRATGGCTACGAAGGC	473	M57602.1
	CMV 1-5 d	CACGGACCGAAGTCCTTCCGAAGAAA		
EAPV	EAPV-IB-1F	CATTGATAATGGCACCTCACC	231	MH922997.1
	EAPV-IB-1R	AGCCAAACTCAAGTCCCTCA		

For PCR diagnosis of DNA viruses (EuLCV, PaLCuGdV), we used GoTaq^®^ DNA Polymerase (Promega, Seoul, Republic of Korea), 2.5mM dNTP, 5× Taq polymerase buffer, and 25mM MgCl_2_. We added 10 ng of DNA extracted from leaves with suspected viral infection, and 10 pmol each of the diagnostic primer sets specific to each virus to the reaction mixture. SimpliAmp^™^ Thermal Cycler (ABI, Thermo Fisher SCIENTIFIC, Seoul, Republic of Korea) was used to perform denaturation for 3 mins at 95°C, followed by 35 cycles of 20 s at 94°C, 30 s at 55°C, and 1 min at 72°C. Finally, the mixture was subjected to final extension for 10 mins at 72°C.

For one-step RT-PCR to diagnose RNA viruses (CMV, EAPV), we used SuPrimescript RT-PCR Premix (Genetbio, Daejeon, Republic of Korea). After adding 10 ng of the extracted RNA and 10 pmol of each primer set to the reaction mixture, we performed the experiment according to the manufacturer’s guidelines. The RT-PCR conditions were 30 mins at 50°C and then 10 mins of reverse transcription at 95°C, followed by 35 cycles of 30 s at 95°C. 30 s at 55°C, and 1 min at 72°C. Finally, the mixture was subjected to a final extension for 5 mins at 72°C.

To check the sizes of the PCR and RT-PCR amplification products, we used 1× TBE buffer and 1.2% agarose gel containing a DNA stain (GoodView^™^, Bratislava, Slovak Republic). We used a 100 bp DNA Ladder (Invitrogen, Carlsbad, CA, USA) and performed electrophoresis for 30 mins at 100 V. Finally, we verified the infection using an image analyzer (WSE-5300 Printgraph CMOS I, Atto, Tokyo, Japan).

### 2.3 Experimental environment and cultivation management

Two experiments were designed to analyze how viral infections affect the quality characteristics of *P. edulis* fruit. The first experiment focuses on the infection and quality characteristics of fruit following viral inoculation at different growth stages (hereafter referred to as the growth stage experiment). The second experiment focuses on analyzing the quality characteristics of fruit by cultivation year (age) following viral infection (hereafter referred to as the cultivation year experiment). The experiments were conducted in a triple-span, double-layer greenhouse located in Jeonbuk State Agricultural Research & Extension Services (JSARES), and the total area was 200 m^2^. The variety of *P. edulis* used in the experiment were Tai-nung No.1 cuttings obtained from the JSARES horticultural department, and 90 plants were planted on April 16th, 2019.

For the growth stage groups, to infect the plants with EAPV and CMV, which can be inoculated via the sap, we planted 45 plants in total, including 15 plants for each virus (5 plants each, in triplicate) and 15 uninfected (control) plants. Here, the data on fruit quality characteristics was used again in the cultivation year experiment. For the cultivation year groups, we planted a total of 45 plants, including 15 saplings each for EAPV and CMV inoculation, and 15 saplings for infection with PaLCuGdV, which was infected mechanically via cutting.

The plants were spaced 2 m × 1 m apart, and we used a T-shaped training method. The minimum winter temperature was maintained at 10°C by warming, and ventilation was provided at high temperatures of 30°C or higher. The experiment period was from April 2019 to October 2022. Fertilizer management and other cultivation techniques were performed in accordance with the *P. edulis* cultivation manual published by the Rural Development Administration (Rural Development Administration, 2019).

### 2.4 Viral infection experiment

In this study, in order to analyze the effects of viral infection on fruit quality characteristics of *P. edulis*, we investigated EAPV, CMV, and PaLCuGdV. One limitation of this study is that we did not include EuLCV. EuLCV is one of the major *P. edulis* viruses in the Republic of Korea, and has been reported to be infected via cuttings or trialeurodes (Jeon et al., 2022). However, because we were unable to obtain specimens infected with EuLCV only, this was excluded from the experiment.

For EAPV and CMV, which can be inoculated via the sap, for virus inoculation and formation of the uninfected group, we planted saplings that had been confirmed healthy by PCR and RT-PCR. The times of virus inoculation for the growth stage experiment were early growth (May), flowering (June), and fruit enlargement (July). Meanwhile, for the experiment on annual changes in quality characteristics with viral infection, we inoculated EAPV and CMV in early growth (May). Using 600 mesh carborundum, we homogenized diseased tissue (v/w, 10/1) in 0.01 M phosphate buffer (pH 7.0), performed sap inoculation, and observed the presentation of disease symptoms. We then verified the infection by RT-PCR.

In the analysis of infection and quality characteristics with viral inoculation by growth stage, the infection rates were converted into percentages by dividing the number of confirmed infected trees by the total number of inoculated trees, and multiplying by 100. Here, because we could not guarantee infection with each virus, we calculated the infection rates after inoculating 5 trees. Next, we analyzed infection and fruit quality characteristics in *P. edulis* with virus inoculation by growth stage, and we analyzed the changes in fruit quality characteristics depending on viral infection status. The viral strains used for inoculation were obtained in the form of freeze-dried infected leaves from the Crop Protection Division of the National Institute of Agricultural Sciences, the Republic of Korea.

For PaLCuGdV, which is infected mechanically during cutting, we verified a single infection with PaLCuGdV using PCR and RT-PCR diagnosis before planting cuttings received from the department of horticulture at JSARES. After planting, we investigated the fruit quality characteristics by cultivation year depending on viral infection status. All experimental groups were isolated using grow tunnels with a mesh size of ≤0.4 mm to prevent contamination between viruses, and the movement of vectors was blocked. For the cultivation method, we adhered to the *P. edulis* cultivation manual (Rural Development Administration, 2019).

### 2.5 Analysis of *P. edulis* fruit quality characteristics with viral infection

After verifying viral infection in each treatment group, we selected three plants each in the EAPV, CMV, and PaLCuGdV treatment groups and the uninfected group over 4 years, from 2019 to 2022. However, in EAPV inoculation in the flowering stage, infection was only confirmed in two individuals, so we only investigated fruit quality characteristics for these two individuals. Meanwhile, in the EAPV early growth and CMV fruit enlargement stages, the infection rates were 0%, meaning that no plants were infected. Therefore, the investigations of fruit quality characteristics were not performed in these stages, and they were not included in the analysis of the results.

In the virus inoculation by growth stage experiment, we investigated the fruit weight, Brix, titratable acidity, marketable fruit rate, and number of fruits per plant. Meanwhile, in the experiment on annual changes in fruit quality characteristics with viral infection, we investigated the fruit weight, Brix, titratable acidity, marketable fruit rate, number of fruits per plant, and the marketable yield per 10 ares. *P. edulis* is a sub-tropical fruit tree that can be harvested in the same year after planting, and is capable of annual flowering and fruit production when the total effective temperature is around 1,500 degree-days (Jeollanamdo Agricultural Research and Extension Services, 2015). Therefore, we cultivated individuals with confirmed viral infections and uninfected individuals in isolation within nets, and we investigated the fruit quality characteristics individually.

The marketable fruit rate was calculated, with reference to the marketable fruit criteria of the Jeollanamdo Agricultural Research and Extension Services (2015), as the percentage of total fruit that were commercially suitable, and this was converted to a marketable yield per 10 ares. For quality characteristics, during the harvesting period in each treatment group (July 20th to September 20th, each year), we analyzed the fruit weight, titratable acidity, and Brix of up to 10 fruit per tree, every day. Analysis was performed immediately after harvesting with no additional ripening period. Brix was measured by diluting 1 g of sample in 10 ml of distilled water, measuring using a digital Brix meter (Atago, PAL-1, Japan), and multiplying by the dilution ratio. Titratable acidity was measured by adding 1% phenolphthalein indicator to 10 ml of sample solution, measuring the volume of 0.1N NaOH required for the sample to reach pH 8.3, and converting to units of tartaric acid.

For the survey method, we adhered to the survey analysis standards for agricultural science and technology (Rural Development Administration, 2012). For the marketable fruit rate, we set marketable fruit criteria for *P. edulis* as fruit weight ≥ 60 g, smooth, purple skin, Brix of ≥16°Bx, and titratable acidity of 2.5–3.0% (Jeollanamdo Agricultural Research and Extension Services, 2015). To analyze the differences in fruit quality characteristics by growth stage in *P. edulis* fruit in each group, to compare annual changes in fruit quality characteristics with viral infection, and to investigate the significance of mean differences over 4 years, we used IBM SPSS Statistics Ver. 21.0 (IBM Co., Armonk, NY, USA) to perform one-way ANOVA with Duncan’s multiple range test for *post-hoc* analysis. Statistical significance was set at α = 0.05.

## 3 Results and discussion

### 3.1 State of viral disease in *P. edulis*

During the survey period, we observed a total of 5 viruses infecting *P. edulis*, and the main viral diseases were PaLCuGdV, CMV, EuLCV, and EAPV (**Table 2**). These results are consistent with previous reports that *P. edulis* cultivation and production are affected by various viruses, and that these are especially important diseases for passion fruit woodiness (Fischer and Rezende, 2008).

**Table 2:** State of viral disease in *P. edulis* in the study region.

Virus	Incidence rate^z^		Occurrence month	Occurrence year		Symptom part^y^
PaLCuGdV	5–80 (32.5)		Jan–Dec	2019–2021		L, Fl, Fr
CMV	0–50 (14.3)		Jan–Dec	2019–2021		L, Fl, Fr
EuLCV	0–80 (14.0)		Jan–Dec	2019–2021		L, Fl, Fr
EAPV	0–60 (10.6)		Jan–Dec	2019–2021		L, Fl, Fr
PLV	0–2 (0.1)	Jan–Dec		2021	L, Fl, Fr	

^z^The incidence rate (%) indicates the minimum to maximum range, and the values in parentheses represent the average incidence rate.

^y^L, Fl, and Fr denote leaf, flower, and fruit, respectively.

Viral diseases lead to very serious injury that greatly reduces the yield of *P. edulis* (Chen et al., 2021). In the Guizhou region of China, the primary viruses detected were EAPV, PLV, and TeMV, with detection rates of 63.65%, 34.7%, and 1.59%, respectively (Ren et al., 2023). Jeon et al. (2022) investigated patterns of viral disease occurrence in *P. edulis* and found that PaLCuGdV had the highest infection rate (68.3%), followed by CMV (58.3%), EuLCV (48.3%), and EAPV (3.3%). The report by Jeon et al. (2022) noted that viral disease occurs persistently in the Republic of Korea, and that the patterns of viral infection are becoming more diverse, showing similar trends to the results of the present study.

The incidence of viral diseases in *P. edulis* is analyzed in **Table 2**. However, in the field, mixed infections of viruses frequently occur. Jeon et al. (2022) reported that the mixed infection rate of *P. edulis* in the Republic of Korea reached 75%, and Choi (2025) stated that the mixed infection rate from 2017 to 2021 was 65.4%. Mixed infections exacerbate symptoms by allowing different viruses to act simultaneously, affecting the replication of each virus (Otsuki and Takebe, 1976). In Taiwan, mixed infection of *P. edulis* with EAPV, EuLCV, and PaLCuGdV resulted in more severe mosaic symptoms on leaves and fruit deformities (Chong et al., 2018). Therefore, the occurrence of mixed infections can further exacerbate economic losses in *P. edulis* cultivation.

**Figure 1:**
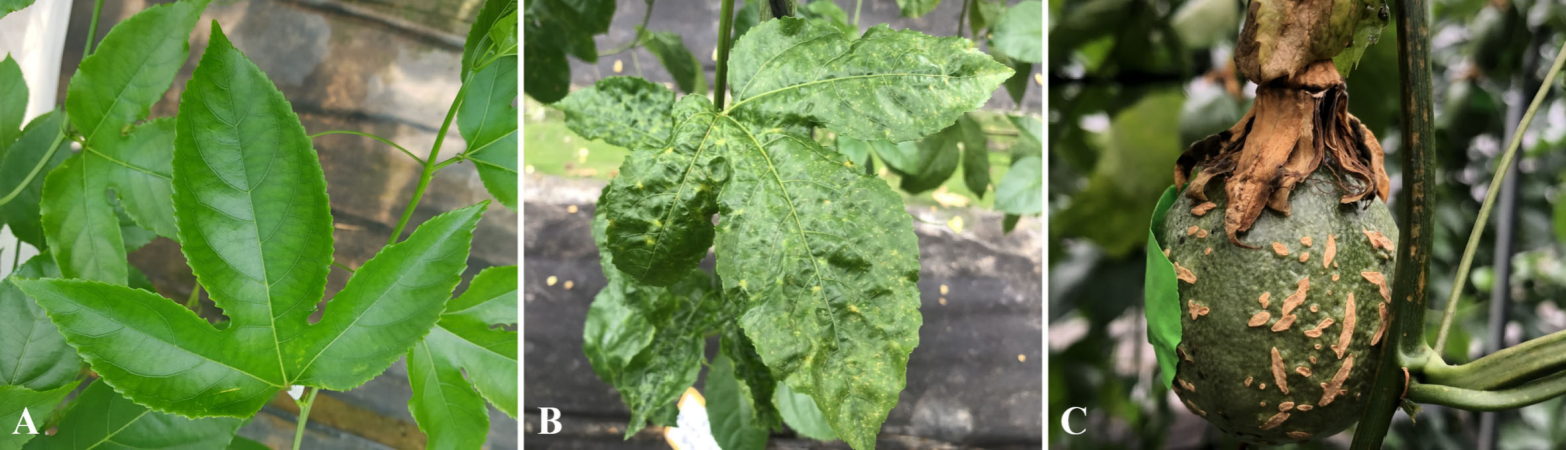
Symptoms of EAPV infection in *P. edulis*. **(A)** Healthy leaves, **(B)** Leaf symptoms after infection with EAPV, and **(C)** Fruit after infection with EAPV. **Supplementary Figure S1** presents the RT-PCR electrophoresis image for EAPV detection.

Although several viruses are involved in major diseases during *P. edulis* cultivation, because processes such as pruning, trimming, and fertilization are usually repeated within plots for 4–5 years of cultivation, EAPV and CMV infections, which can spread via sap, showed increasing incidence with cultivation years (**Table 3**). EAPV is one of the viruses in the genus *Potyvirus*, known for its wide host range infecting *P. edulis* fruit, and is primarily transmitted by aphids in a non-persistent manner (Wu et al., 2024). In contrast, CMV is a representative virus of the genus *Cucumovirus*, known for its wide host range and strong transmissibility, and is also transmitted by aphids (Chen et al., 2021). In particular, EAPV causes significant economic loss because it reduces production and marketable yield through serious deterioration of tree vitality, corking of stems and fruit, wrinkling, mottling, and mosaic symptoms on leaves, and fruit deformation (Chong et al., 2018; **Figure 1**).

**Table 3:** Analysis of correlations between *P. edulis* cultivation years and incidence rates (%) of major viruses.

Cultivation years (years)	EAPV	EuLCV	CMV	PaLCuGdV
1	3.2	5.8	7.8	23.5
2	4.8	9.4	8.8	21.1
3	4.5	15.0	8.8	36.7
4	10.7	2.7	4.5	25.5
5	30.0	37.1	41.4	55.7
Mean (r)	10.6 (0.47^**^)	14.0 (0.42^**^)	14.3 (0.41^**^)	32.5 (0.32^*^)

Asterisk indicates level of significance: *p<0.05, **p<0.01.

During the study period, samples were collected from a plot in Iksan in 2021 showing leaf curling, yellowing, stunting, and fruit malformation, and after next-generation sequencing (NGS), RT-PCR, and pathogenicity testing, the pathogen was finally identified as Passiflora latent virus (PLV) of the genus Calavirus, marking the first report of this virus in the Republic of Korea (Choi and Ju, 2023). PLV can be transmitted via sap and diseased saplings. In addition, Cho et al. (2021) reported a case of asymptomatic PLV infection in persimmon (*Diospyros kaki*) in the Republic of Korea, indicating the need for further surveys to investigate its potential spread to surrounding crops.

### 3.2 Correlations between *P. edulis* cultivation year and incidence rates of major viruses

When we analyzed the correlations between cultivation year (age) of *P. edulis* and the incidence rates of various viruses, we found that increasing cultivation years (i.e., older age) showed a significant positive correlation with increasing incidence rates. EAPV especially showed a high incidence rate of 30.0% in 5-year-old *P. edulis*, and showed the strongest correlations (r=0.47**). This was followed by EuLCV (r=0.42**), CMV (r=0.41**), and PaLCuGdV (r=0.32*; **Table 3**).

In *P. edulis* cultivation, viral diseases can severely impair productivity, and the useful lifespan of *P. edulis*, which is typically around 5 years, can be shortened by up to 1 year due to disease. For this reason, nomadic cultivation patterns have been observed for this crop in some regions of Brazil (Fischer and Rezende, 2008).

### 3.3 Infection and fruit quality characteristics with EAPV inoculation by *P. edulis* growth stage

We inoculated EAPV to the sap of *P. edulis* at different growth stages and investigated the infection rates. During early growth, 25 days after planting, even when EAPV was inoculated to young leaves, we did not observe infection even up to 90 days after the date of inoculation. However, we observed infection rates of 40% in the flowering stage 55 days after planting, and 60% when inoculated during the fruit enlargement stage (**Table 4**).

**Table 4:** Analysis of infection rates and fruit quality characteristics according to EAPV inoculation at different growth stages.

Growth stage (Inoculation date)	Infection rate (%)	Fruit weight (g)	Brix (°Bx)	Titratable acidity (%)	Marketable fruit rate (%)	No. of fruits per plant
Uninfected	0.0	80.8 a^z^	17.8 b	2.78 a	76.8 b	40.3 a
Early growth (May 10, 2019)	0.0	–	–	–	–	–
Flowering (June 13, 2019)	40.0	80.4 a	17.2 a	3.04 b	60.5 a	42.5 b
Fruit enlargement (July 15, 2019)	60.0	83.3 a	17.5 ab	2.91 ab	67.0 ab	39.3 a
F (p)	–	2.981 (0.141)	8.599 (0.24)	4.428 (0.78)	6.476 (0.041)	7.935 (0.028)

^z^Duncan’s multiple range test (α=0.05), a<b.

The time required for disease symptoms to appear decreased from 53 days at 28°C during the flowering stage to 25 days at 30°C in the fruit enlargement stage (data not shown), indicating that temperature significantly influences virus replication. EAPV, classified under the genus *Potyvirus*, behaves similarly to potato virus Y, where Choi et al. (2017) reported systemic infection times decreasing from 14 days at 20°C to 5.7 days at 28°C. These findings suggest that lower temperatures, such as 20°C during early growth, reduce replication rates and hinder the virus in overcoming plant defenses.

Infection with EAPV during the flowering stage led to worse fruit quality in *P. edulis*, with lower Brix and higher titratable acidity, significantly reducing marketable fruit rates compared to healthy fruit. In contrast, infections during the fruit enlargement stage resulted in similar fruit quality metrics to uninfected plants, but marketable fruit rates still decreased significantly (p=0.041; **Table 4**). Additionally, early growth stage inoculation led to poor marketable fruit rates, likely due to stress from physical inoculation affecting tree vigor and rooting.

### 3.4 Infection and changes in fruit quality characteristics following CMV inoculation in different *P. edulis* growth stages

We inoculated CMV into the sap of *P. edulis* at various growth stages. Infection rates were 60% during early growth (30 days after planting) and 80% during flowering (60 days after planting), with no infection during the fruit enlargement stage (105 days after planting) (**Table 5**). The time required for symptoms to appear was 27 days at 20°C during early growth and 30 days at 25°C during flowering (data not shown).

**Table 5:** Infection rates and changes in fruit quality characteristics following CMV inoculation in different *P. edulis* growth stages.

Growth stage (Inoculation date)	Infection rate (%)	Fruit weight (g)	Brix (°Bx)	Titratable acidity (%)	Marketable fruit rate (%)	No. of fruits per plant
Uninfected	0.0	80.8 ab^z^	17.8 b	2.78 b	76.8 a	40.3 c
Early growth (May 17, 2019)	60.0	76.7 a	17.0 a	2.58 a	72.6 a	32.0 a
Flowering (June 18, 2019)	80.0	84.2 b	17.7 b	2.72 ab	73.0 a	37.7 b
Fruit enlargement (July 30, 2019)	0.0	–	–	–	–	–
F (p)	–	7.381 (0.24)	27.697 (0.001)	3.40 (0.103)	0.155 (0.860)	44.455 (<0.001)

^z^Duncan’s multiple range test (α=0.05), a<b<c.

Ko et al. (2009) inoculated zucchini yellow mosaic virus (ZYMV) to cucumbers at different growth stages, and reported that, when inoculated at ratios of 25%, 50%, and 100% during semi-forced cultivation in April to June, when the aphid density is high and temperatures are rising, the initial incidence rates started at 6.9%, 23.7%, and 48.7%, respectively, and continually increased until the later stages of growth, when all eventually showed a 100% incidence rate. Meanwhile, it has been reported that a spinach strain of CMV did not proliferate or cause symptoms in *Nicotiana* spp. at 30°C or higher, and that the count of aphids, which act as vectors, increases at certain temperatures in the range 20–25°C (Yin et al., 2024). When CMV was inoculated to *P. edulis* at different growth stages, the results were similar. Our findings align with these, showing high CMV infection rates in *P. edulis* during early growth and flowering stages, with no infection during fruit enlargement when temperatures were high (**Table 5**).

When we analyzed fruit quality characteristics, fruit that had been infected in the early growth stage showed significantly lower Brix (0.8°Bx) and titratable acidity (0.3%) compared to uninfected fruit, and a lower mean number of fruits per plant (8.3). In the flowering stage, the mean number of fruits per plant (2.6) was lower than uninfected fruit, but the fruit weight was heavy (3.4 g), and there were no significant differences in other quality characteristics (**Table 5**).

### 3.5 Changes in *P. edulis* fruit quality characteristics with viral infection by cultivation year

Compared to healthy *P. edulis*, virally infected plants showed a decline in marketability with increasing cultivation year (age), with significant decreases in Brix and number of fruits per plant. When infected with EAPV, older age was associated with a mean decrease of 36.3 fruits per plant compared to the uninfected group, the fruit weight was lighter, and the Brix also decreased, resulting in a decline of 26.8%p in the marketable fruit rate (**Table 6**). Notably, the external appearance showed symptoms such as fruit corking and malformation, resulting in the loss of commercial value (**Figure 1C**). Iwai et al. (2006) reported that EAPV symptoms include vein necrosis and rugosity of the upper trifoliate leaves, misshapen, woody and pitted fruit, and stunted vegetative growth. We also found that *P. edulis* infected with EAPV showed more severely stunted growth with increasing cultivation year, suggesting that this could have negative effects on growth and development. According to previous studies, infection by TeMV, a member of the genus *Potyvirus* like EAPV, resulted in a 21.6% reduction in total fat content in *P. edulis* fruit, and levels of total acid and vitamin C were also lower compared to healthy fruit. In contrast, the total phenolic content, a secondary metabolite involved in host-pathogen interactions, increased by 19.1%. These findings suggest that viral infection can selectively modulate the chemical composition of plants, supporting the results of this study (Chen et al., 2018).

**Table 6:** Comparison of *P. edulis* fruit quality characteristics following viral infection by cultivation year.

Classification	Year	Fruit weight (g)	Brix (°Bx)	Titratable acidity (%)	Marketable fruit rate (%)	No. of fruits per plant	Marketable yield (kg/10a)
EAPV	1	82.5 a^z^	17.4 ab	2.97 a	63.5 a	39.8 a	479.2
	2	61.9 a	14.7 a	2.64 b	31.0 a	66.0 a	290.7
	3	74.4 b	14.5 a	3.56 b	45.2 a	62.0 a	483.9
	4	66.8 ab	14.2 a	2.92 b	65.2 a	66.7 a	667.1
	Mean	72.0 ab	15.3 a	3.01 b	53.9 a	57.6 a	480.2
CMV	1	82.7 a	17.5 ab	2.73 a	69.0 ab	37.3 a	482.4
	2	55.8 a	15.2 a	2.28 a	39.4 a	93.0 a	463.7
	3	57.7 a	15.5 ab	2.57 a	54.9 ab	81.0 ab	589.9
	4	64.7 a	15.6 ab	2.69 a	73.6 ab	87.3 b	921.1
	Mean	66.9 a	16.1 ab	2.60 a	61.6 ab	72.2 ab	614.3
PaLCuGdV	1	81.3 a	17.2 a	2.87 a	64.0 a	48.1 b	576.8
	2	84.3 c	15.5 ab	2.66 b	56.6 b	86.6 a	954.1
	3	81.9 bc	15.3 ab	3.06 ab	65.5 b	73.0 a	904.9
	4	77.3 c	15.2 ab	3.19 a	79.0 b	86.7 b	1214.3
	Mean	81.2 c	15.8 ab	2.94 b	66.3 b	73.6 ab	912.5
Control (uninfected)	1	80.8 a	17.8 b	2.78 a	76.8 b	40.1 a	544.9
	2	75.9 b	16.3 b	2.73 b	78.2 c	129.7 b	1779.6
	3	84.2 c	16.3 b	2.58 a	83.4 c	96.3 b	1555.6
	4	72.3 bc	16.2 b	3.15 a	84.5 b	109.7 c	1544.3
	Mean	78.3 bc	16.7 b	2.82 ab	80.7 c	93.9 b	1356.1
F (p)	1	2.513	3.427	2.028	2.783	8.347	–
		(0.132)	(0.073)	(0.189)	(0.110)	(0.008)	
	2	39.296	8.156	7.639	27.95	12.53	–
		(<0.001)	(0.015)	(0.018)	(0.001)	(0.005)	
	3	22.769	4.266	5.37	18.505	6.772	–
		(0.001)	(0.062)	(0.039)	(0.002)	(0.024)	
	4	10.719	1.447	13.81	5.425	22.961	–
		(0.004)	(0.300)	(0.002)	(0.025)	(<0.001)	
	Mean	7.039	1.713	4.314	10.319	4.018	–
		(0.001)	(0.180)	(0.010)	(<0.001)	(0.014)	

^z^Duncan’s multiple range test (α=0.05), a<b<c.

**Figure 2:**
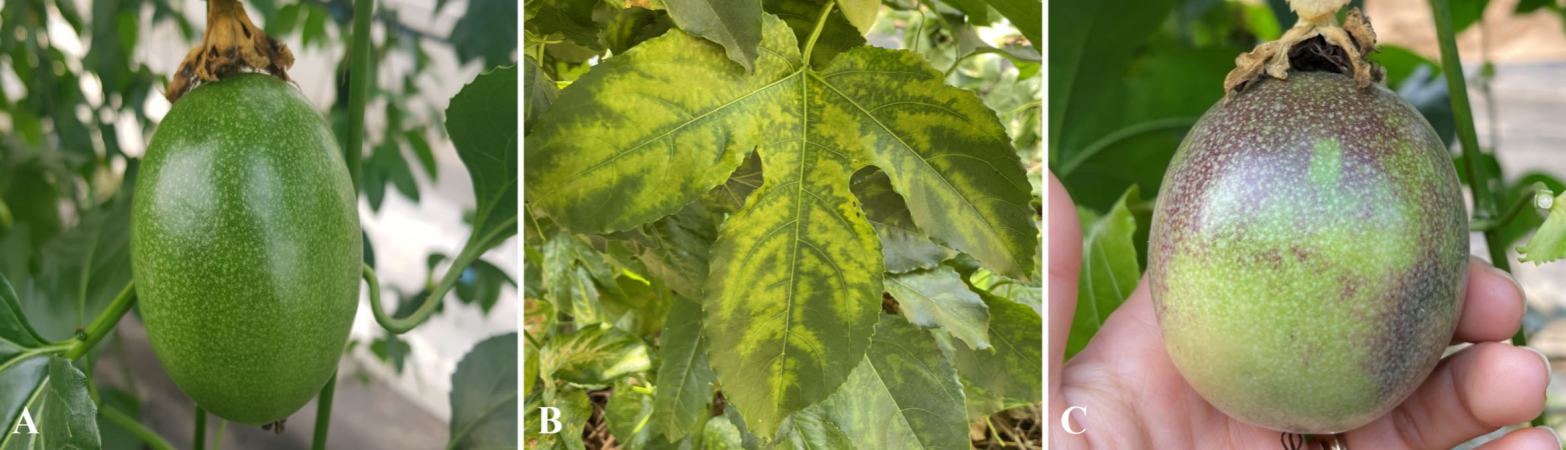
Symptoms of CMV infection in *P. edulis.* **(A)** Healthy *P. edulis* fruit, **(B)** Leaf symptoms after infection with CMV, and **(C)** Fruit after infection with CMV. **Supplementary Figure S2** presents the RT-PCR electrophoresis image for CMV detection.

*P. edulis* infected with CMV exhibited a significant decrease in fruit weight with increasing cultivation years, resulting in a 19.1%p reduction in marketable fruit rate compared to uninfected plants (**Table 6**). The infection also led to more pronounced symptoms, such as clear spots, yellowing, and mosaic patterns on leaves, along with yellowing and pigmentation defects on the fruit, diminishing commercial value (**Figure 2**). These findings align with previous studies, which reported CMV-infected *P. edulis* showing mosaic patterns, necrosis, leaf curl, decreased chlorophyll content, and downregulation of genes related to photosynthesis and chloroplasts (Yeturu et al., 2018; Chen et al., 2021; Zhao, 2021).

*P. edulis* infected with PaLCuGdV did not show significant differences with the uninfected group in terms of external appearance or fruit quality characteristics, and the mean fruit weight was heavier, at 2.9 g. However, compared to the uninfected group, the mean number of fruits per plant was lower by 20.3, and the titratable acidity was higher, resulting in a mean decrease of 32.7% in marketable yield (**Table 6**).

PaLCuGdV is in the genus *Begomovirus*, and there have been relatively few studies of *Begomovirus* infection in *P. edulis* compared to *Potyvirus* spp. This implies that the commercial cultivation of *P. edulis* is less affected currently by *Begomovirus* spp (Wu et al., 2024). Tree vigor was managed by identical pruning and trimming processes, in accordance with a standard cultivation manual (Rural Development Administration, 2019), in all treatment groups, but we still observed annual differences in the numbers of *P. edulis* fruits. Nevertheless, the years with the highest and lowest yields were the same in all treatment groups, and the damage due to viral infection in *P. edulis* was most severe for EAPV, followed by CMV, then PaLCuGdV.

The demand for *P. edulis* is increasing due to its unique flavor, prompting expanded cultivation. However, the plant is vulnerable to viral diseases that can significantly reduce yield and, in severe cases, cause complete cultivation failure. In the Republic of Korea, *P. edulis* was introduced from Taiwan, where cultivation areas decreased dramatically from 1,392 ha in 1982 to less than 100 ha in 1990 due to viral diseases (Chen, 1991; Wu et al., 2024). The introduction brought previously unreported viruses like EAPV, EuLCV, and PaLCuGdV. Healthy, uninfected *P. edulis* demonstrates excellent quality, stable fruit numbers, and a high marketable fruit rate over 80%, with sapling replanting typically occurring at 4–5-year intervals. Proper prevention and management of viral diseases may allow cultivation beyond 5 years.

## 4 Conclusions

This study analyzed major viral diseases in *P. edulis* in the Republic of Korea and assessed the effects of viral infection on fruit quality and productivity. This problem directly affects the commercial value of *P. edulis* and the income of farms, and our study provides new insights that could help with the development of strategies for the prevention and management of viral diseases in *P. edulis*.

In particular, we demonstrated that the incidence rates of viral diseases due to EAPV, EuLCV, CMV, and PaLCuGdV increase with increasing cultivation years. Therefore, as cultivation years increase, *P. edulis* growing farms need to implement even more thorough preventive management of viral diseases. In addition, we demonstrated that infection with EAPV, CMV, and PaLCuGdV have significant effects on the fruit quality and productivity of *P. edulis*. Infection with EAPV especially could lead to serious economic losses for farms as it can cause a large decrease in the marketable fruit rate. These findings provide important guidelines for disease management in *P. edulis*.

Based on these findings, *P. edulis* growers should select virus-free seedlings when renewing plants and remove weeds and pruning residues around cultivation areas to eliminate sources of infection. Choi (2025) particularly highlighted that mixed infections with EuLCV exacerbate symptoms and fruit deformities in *P. edulis* grown in the Republic of Korea, emphasizing the need for focused management of specific weeds from the Asteraceae and Solanaceae families around cultivation sites to prevent spread. Additionally, to prevent vectors, growers should conduct trap monitoring and apply chemical control, as well as thoroughly disinfect tools during operations. These specific management strategies will not only be effective in preventing viral diseases in *P. edulis* but also help minimize economic losses for growers.

This study has some limitations. Primarily, the survey range was restricted to a specific region, and we were unable to acquire plants infected with EuLCV alone, which posed challenges for data collection. Future studies should conduct additional surveys of disease incidence patterns in diverse regions and environmental conditions, enabling the development of more generalized management strategies.

Nevertheless, our study provides foundational data to increase the stability of *P. edulis* cultivation and prevent the spread of viral disease, thereby helping to improve its practical applicability in actual agricultural settings. These results are expected to play a key role in improving the sustainability of the *P. edulis* industry and promoting economic stability for farms.

## Data Availability

The raw data supporting the conclusions of this article will be made available by the authors, without undue reservation.
