# Supplementary material for: Effects of viral infection on passion fruit (Passiflora edulis) quality and productivity in the Republic of Korea
Source: Front Plant Sci. 2025 Jun 19;16:1612094. doi: 10.3389/fpls.2025.1612094 (PMC12222072; doi:10.3389/fpls.2025.1612094)
Supplement: Supplementary file 2 [file DataSheet2.pdf]

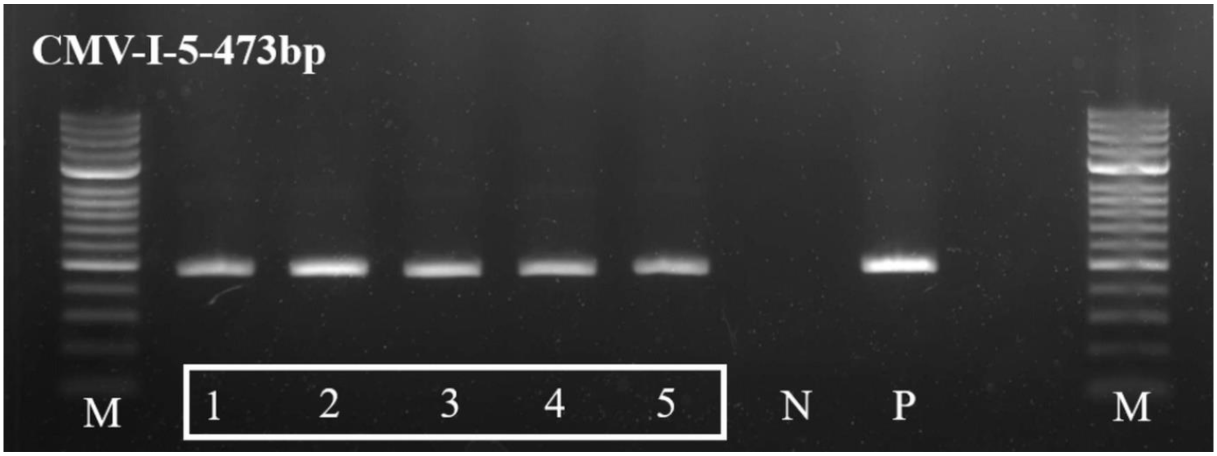

Supplementary Figure 2. Detection of cucumber mosaic virus (CMV) by RT-PCR using specific primer pairs. Lane M: 100 bp ladder; lanes 1–5: symptomatic plants showing positive amplification; lane N: non-symptomatic plant used as a negative control; lane P: symptomatic plant used as a positive control. A specific 473 bp DNA fragment was successfully amplified from *Passiflora edulis* infected with CMV.
